# Evaluation of a combination adherence strategy to support HIV antiretroviral therapy for pregnancy and breastfeeding in Malawi: A pilot randomized clinical trial

**DOI:** 10.1371/journal.pone.0319735

**Published:** 2025-04-28

**Authors:** Wilbroad Mutale, Lauren A. Graybill, Friday Saidi, Twambilile Phanga, K. Rivet Amico, Kellie Freeborn, Nora E. Rosenberg, Lauren M. Hill, Twaambo Hamoonga, Brian D. Richardson, Katie R. Mollan, Benjamin H. Chi

**Affiliations:** 1 Department of Health Policy and Management, University of Zambia, Lusaka, Zambia; 2 Institute for Global Health and Infectious Diseases, University of North Carolina at Chapel Hill, Chapel Hill, North Carolina, USA; 3 UNC Project Malawi, Lilongwe, Malawi; 4 Department of Health Behavior and Health Education, University of Michigan, Ann Arbor, MI, United States of America; 5 Department of Obstetrics and Gynecology, University of North Carolina at Chapel Hill, Chapel Hill, North Carolina, United States of America; 6 Department of Health Behavior, University of North Carolina at Chapel Hill, Chapel Hill, North Carolina, United States of America; 7 Department of Population Studies and Global Health, University of Zambia, Lusaka, Zambia; 8 UNC Center for AIDS Research, University of North Carolina at Chapel Hill, Chapel Hill, North Carolina, United States of America; Medical Research Council, SOUTH AFRICA

## Abstract

**Background:**

There has been tremendous progress in reducing vertical transmission of HIV in the past two decades due to the broad availability of antiretroviral therapy (ART) globally. Despite this progress, new paediatric infections are still occurring.

**Methods:**

In a pilot study, we evaluated a combination adherence support package, which included an adapted motivational interviewing-informed counselling approach (Integrated Next Step Counselling, iNSC) and an optional adherence supporter, for pregnant and breastfeeding women living with HIV. Participants were recruited from the antenatal clinic in Lilongwe, Malawi. Eligible participants were randomly allocated 1:1 to receive either the combination adherence package (intervention) or standard care (control) at the health facility. Our clinical outcome, measured at three- and six-month follow-up, was a composite endpoint of study retention with HIV viral suppression (HIV RNA <40 copies per mL).

**Results:**

We screened 106 women living with HIV between March and July 2020. Of these, 100 women enrolled and were randomly assigned to intervention (n=51) or control (n=49). The majority of participants (94 of 100; 94%) were newly diagnosed with HIV. Retention in care was 92% at three months and 84% at six months. Three-quarters of women retained in care were virally suppressed at the three- and six-month study visits. At three months, our composite outcome (retention & viral suppression) was achieved by 70.6% (36/51) and 69.4% (34/49) of women in the intervention and control groups, respectively. At six months, this composite outcome was achieved by 68.6% (35/51) of the intervention group and 61.2% (30/49) of the control group (probability difference: 7.4%, 95% CI: -11.3%, 26.1%).

**Conclusion:**

These encouraging pilot findings suggest that this combination adherence package could be used to support ART adherence among pregnant and breastfeeding women living with HIV. We demonstrate feasibility of using a combined measure of adherence and viral suppression as an outcome measure.

**Trial registration:**

ClinicalTrials.gov (NCT04330989).

## Introduction

We have seen tremendous progress in reducing vertical transmission of HIV in the past two decades. The HIV prevention programs have expanded rapidly resulting in reduction in incident paediatric infections globally. [1,2] Despite this progress, breakthrough paediatric infections are still being recorded and currently there are global efforts to eliminate paediatric infection especially with the universal availability of antiretroviral drugs in low and middle income countries (LMICs) which are effective in reducing the risk of vertical acquisition of HIV [3–5].

Without antiretroviral treatment, the risk of HIV acquisition for children is 15% to 30% during gestation or labour, with an additional 10% to 20% risk associated with extended breastfeeding. [6] In contrast, the risk drops to less than 5% with ART during pregnancy and breastfeeding with optimal adherence [7].

ART adherence and retention can be challenging over the course of pregnancy and breastfeeding, especially following childbirth. [8,9] It has been reported that up to 20% of pregnant women living with HIV drop out of care in the first six months on ART and for those who continue, ART adherence can vary significantly, leaving potential for unsuppressed viral load that increases the risk of vertical HIV transmission. [10] Early studies suggested that women often drop out of care during the postpartum period due to various individual, social and system barriers [11,12].

Several studies have looked at facilitators and barriers to ART adherence in pregnancy and postpartum. These barriers can be divided into individual, interpersonal and health system barriers. Individual-level barriers include personal understanding of HIV, ART, and prevention of mother to child transmission of HIV (PMTCT) and personal difficulties in managing practical demands of taking ART.

Barriers at an interpersonal level include partner support and difficulty to disclosure [13]. These barrier perpetuate stigma at a community level. [14] At health system level main structural barriers and enablers are related to ease of health system utilisation and health worker attitudes. [13,15,16] Various Interventions are being tested to address these barrier Interventions that use of technology, behavioural interventions and system re-structuring [17–19].

Healthcare systems can be improved to better assist women navigating complex issues of handling HIV diagnosis and health system and social barriers. For example, providing opportunities for support of women’s wellbeing and mental health as they navigate ART, may be a meaningful shift from traditional instructions-based approaches that do not give women space to explore or identify meaningful strategies of their own.

To address barriers described, we designed a combination adherence support package which included an adapted motivational interviewing-informed counselling approach (Integrated Next Step Counselling, iNSC) and an optional adherence supporter, for pregnant and breastfeeding women living with HIV. Here, we describe the feasibility and potential impact of the combination adherence intervention on retention and viral suppression outcomes among pregnant and breastfeeding women in Lilongwe, Malawi. This is part of a larger study initiative studying an HIV “status neutral” approach to health services for pregnant and breastfeeding women, across the continuum of prevention, care, and treatment.

## Methods

### Study design

This was a pilot randomised clinical trial to assess the combination approach to support ART adherence. We adapted a motivation interviewing-informed brief counselling approach iNSC [21] for pregnant and breastfeeding women living with HIV. Details of the methodology are published in a protocol paper elsewhere. [20] Below we summarise the intervention and procedures.

### Intervention

The Tonse Pamodzi adherence intervention comprised two parts: (1) iNSC adapted for well-being and ART adherence support for pregnant and breastfeeding women, and (2) voluntary inclusion of an adherence supporter.Those in the intervention arm were asked to identify an adherence supporter from their own social networks. *Omukhulupilira* (“trusted one” in Chichewa) could include a partner, family member, or friend who could provide emotional, instrumental, and informational social support. Selecting such a supporter was recommended, but not mandated. Once nominated, an Omukhulupilira received an in-person orientation on how to provide positive support to the participant. Participants received the iNSC intervention while the control participants received the standard of care described below.Participant visits occured at enrolment and at 1, 3 and 6 months time points. These were seen by qualified nurses who administered the intervention. Study procedures for each visit included: Collection of blood samples and collection of urine specimens. The laboratory tests conducted included: HIV RNA (viral load), haemoglobin, syphilis screening and urine dipstick. These specimens were collected for safety monitoring and outcome measurement. All testing procedures followed local standard guidelines as prescribed by the Ministry of Health in Malawi.

### Details of these intervention components are described elsewhere. [20,22]

#### Current standard of care.

Participants assigned to the control group received standard counseling in PMTCT, safe obstetrics, and newborn care, based on the Malawi Ministry of Health guidelines. [23] In addition, all participants received education regarding HIV, including antiretroviral therapy drug safety, adherence, and potential side effects.

### Recruitment and retention procedures

#### Screening, inclusion, exclusion criteria.

Participants were recruited from Bwaila District Hospital which is the largest referral hospital in Lilongwe, Malawi. We enrolled women living with HIV who were 18 years of age or older and had a documented pregnancy by urine pregnancy test or physical exam. All had initiated on first-line ART within the past 30 days, either for the first time or after treatment interruption of 6 months or longer. Candidates were screened for intimate partner violence (IPV) and social harms; those at risk were excluded from further enrollment. All participants underwent informed consent before study procedures began.

#### Randomization.

Eligible participants were randomly allocated 1:1 to receive either the combination adherence package (intervention) or standard care at the health facility (control)

#### Study activities.

After enrollment participants returned at one, three, and six months. The following tests were performed at the visits included HIV RNA (viral load), haemoglobin, syphilis screening (if not available in the antenatal record), and urine dipstick. HIV viral load was measured on-site at the UNC Project-Malawi Central Laboratory (Lilongwe, Malawi). ART dispensation was coordinated with the local HIV services at the study facility. Typically, ART was dispensed every 3 months, however ART prescription intervals were extended to every 6 months due to COVID-19 restrictions. We collected data on the prescribed ART drugs, pharmacy dispensations, and adherence evaluations at short visits every month, timed with routine clinic visits. In the intervention arm, women received the intervention counseling in addition to standard of care at each study visit. While women in the control arm only received the standard of care.

#### Retention and viral load outcomes.

Our primary outcome, measured at three and six months, was a composite measure of study retention with HIV viral suppression. Retention was measured using information on study visit attendance. Women who presented for their visit within the pre-specified visit window were classified as retained while those who missed their visit, or presented for their visit outside the visit window, were classified as not retained. Window periods were define as follows: M0 to M1 - plus or minus 2 weeks (14days) from target date. M1 to M3 - plus or minus 4 weeks (30days) from target date M3 to M6 - plus or minus 8 weeks (60days)from target date. Our measure of HIV viral suppression was based on the detection of HIV RNA in plasma samples collected at each study visit. Women with <40 copies/mL were classified as virally suppressed at that study visit while women with ≥40 copies/mL were classified as unsuppressed. At each assessment point, women retained in the study with viral suppression met our main clinical outcome. Women not retained in the study, as well as women retained in the study with detectable levels of HIV RNA in their plasma samples, were considered outcome failures. Secondarily, we assessed retention and viral suppression separately among women retained in the study at three and six months.

#### Analytic approach.

At both three and six months, we compared the proportion of women retained with HIV viral suppression between intervention and control groups using a probability difference (PD), analogous to a risk difference, and corresponding Wald 95% confidence interval (CI) This approach was also used to compare each component of our primary clinical endpoint—retention and HIV viral suppression among those retained in the study—between randomization groups at each time point. In sensitivity analyses, we used inverse probability of treatment weights to adjust for imbalances in baseline covariates that were expected to be associated with retention and HIV viral suppression (continuous measure of log(10) HIV RNA viral load, gestational age, income source, and number of children). Inverse probability of treatment weighting (IPTW) is used to adjust for confounding in observational studies. IPTW uses the propensity score to balance baseline patient characteristics in the exposed and unexposed groups by weighting each individual in the analysis by the inverse probability of receiving his/her actual exposure. [24].

All analyses were conducted using Windows SAS version 9.4 (SAS Institute, Cary, NC, USA) and R version 4.1.1.

#### Sample size considerations.

Despite its randomized design, this pilot trial was not powered to detect differences in these clinical outcomes (i.e., retention, viral suppression). Our planned sample size of n=100 was chosen based on operational considerations including recruitment feasibility and study resources.

#### Ethical review.

Our study protocol was approved by the Malawi National Health Services Research Committee (Lilongwe, Malawi; protocol 19/05/2334) and the University of North Carolina at Chapel Hill Institutional Review Board (Chapel Hill, NC, USA, protocol 19–1060).

## Results

**Fig 1:**
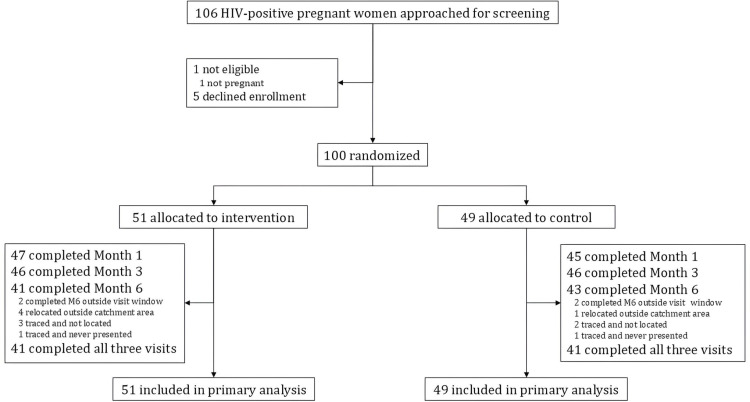
Randomization assignments were generated independently by the study statistician (KRM) using a permuted block design. Staff not otherwise involved in the study procedures placed the randomization assignments in sequentially numbered, opaque, sealed envelopes. The envelopes were opened sequentially for each new enrollment.

Between March 2020 to July 2021, we screened 106 women who were living with HIV-and attending antenatal care at Bwaila District Hospital for study participation. Of these, 100 women enrolled and were randomly assigned 1:1 to either the intervention group (n=51) or control group (n=49) (Fig 1: Consort flow Diagram). The majority of participants (94 of 100; 94%) reported being newly diagnosed with HIV and had initiated ART in the 30 days prior to study enrollment. Baseline characteristics for the study population are presented in Table 1. Notably, the proportion of women virally suppressed at baseline was higher in the intervention group (7 of 51; 14%) than in the control group (3 of 49; 6%).

**Table 1:** Baseline characteristics of HIV-positive pregnant women enrolled in the Tonse Pamodzi Study.

		Intervention Group (n=51)	Control Group (n=49)	Total (n=100)
Age at enrollment (years)	Median (Q1, Q3)	26 (23,30)	27 (24,33)	26 (23,31)
Primary school complete	Yes	29 (57%)	28 (57%)	57 (57%)
Electricity in home	Yes	13 (25%)	13 (27%)	26 (26%)
Running water in home	Yes	10 (20%)	12 (24%)	22 (22%)
Partner is primary source of money	Yes	27 (53%)	21 (43%)	48 (48%)
Time to clinic (minutes)	Median (Q1, Q3)	34 (30, 45)	35 (30, 45)	35 (30, 45)
Gestational age at enrollment (weeks)	Median (Q1,Q3)	26 (20,29)	24 (19,28)	24 (20,28)
	First trimester	2 (4%)	2 (4%)	4 (4%)
	Second trimester	30 (59%)	34 (69%)	64 (64%)
	Third trimester	19 (37%)	13 (27%)	32 (32%)
Gravidity	No prior pregnancies	12 (24%)	8 (16%)	20 (20%)
Number of living children	Median (Q1, Q3)	1 (0, 2)	2 (1, 2)	1 (1, 2)
	No living children	13 (25%)	12 (24%)	25 (25%)
	1-2 living children	31 (61%)	28 (57%)	59 (59%)
	3+ living children	7 (14%)	9 (18%)	16 (16%)
Prior ART user	Yes	2 (4%)	4 (8%)	6 (6%)
Viral load at enrollment (log10 copies/mL)	Median (Q1, Q3)	4 (2,4)	4 (3,5)	4 (3, 4)
	Not Suppressed (≥40 copies/mL)	44 (86%)	46 (94%)	90 (90%)
	Suppressed (<40 copies/mL)	7 (14%)	3 (6%)	10 (10%)
Consumed alcohol during current pregnancy	Yes	1 (2%)	1 (2%)	2 (2%)
Number of lifetime sex partners	1 lifetime sex partner	10 (20%)	14 (29%)	24 (24%)
	2-3 lifetime sex partners	32 (63%)	25 (51%)	57 (57%)
	4+ lifetime sex partners	9 (18%)	10 (20%)	19 (19%)
Primary partner age (years)^^^	Median (Q1,Q3)	31 (28,37)	33 (29,36)	33 (28,36)
Relationship length (years)^^^	Median (Q1,Q3)	3 (1,5)	3 (1,7)	3 (1,6)
Residing with primary partner^^^	Yes	44 (90%)	44 (96%)	88 (93%)
Married to primary partner^^^	Yes	47 (96%)	45 (98%)	92 (97%)
Number of sexual intercourse acts in the past 30 days^^^	Median (Q1,Q3)	8 (1,10)	8 (4,12)	8 (3,12)
Condom use with primary partner in past 30 days^*^	Never	30 (83%)	38 (90%)	68 (87%)
	Sometimes	2 (6%)	3 (7%)	5 (6%)
	Consistent	4 (11%)	1 (2%)	5 (6%)
Primary partner HIV status^^^	HIV-negative	10 (20%)	9 (20%)	19 (20%)
	HIV-positive	10 (20%)	8 (17%)	18 (19%)
	Partner never tested for HIV	5 (10%)	5 (11%)	10 (11%)
	I don’t know	24 (49%)	24 (52%)	48 (51%)
Disclosed HIV status to primary partner^^^	Yes	27 (55%)	28 (61%)	55 (58%)

^ Among women who reported at least one sex partner in the past three months.

* Among women who reported at least one sex act in the past 30 days (n=78).

### Retention and adherence outcomes.

Overall, 92 of 100 women (92%) completed a three-month study visit and 84 of 100 women (84%) completed a six-month visit. Retention was not different between intervention and control groups at three months (90.2% vs. 93.9%; PD: -3.7%, 95% CI: -14.2%, 6.9%) and six months (80.4% vs. 87.8%; PD: -7.4%, 95% CI: -21.6%, 6.9%). At each time point, estimates from our adjusted sensitivity analyses were closer to the null value.

Approximately three-quarters of women retained in the study were virally suppressed at the three- and six-month study visits. At three months, 78.3% of women in the intervention group and 73.9% of women in the control group were virally suppressed (PD: 4.3%, 95% CI: -13.1%, 21.8%). At six months, 85% of women in the intervention group and 69.8% of women in the control group were virally suppressed (PD: 15.6%, 95% CI: -1.9%, 33.1%). At both three and six months, differences between intervention and control groups were attenuated after adjusting for imbalanced baseline covariates.

When we considered our composite outcome, study retention with viral suppression, there was no difference between the intervention and control groups at three months (70.6% vs. 69.4%, PD: 1.2%, 95% CI: 16.8%, 19.2%). At six months, we observed a small difference, but the point estimate was imprecise (68.6% vs. 61.2%, PD: 7.4%, 95% CI: -11.3%, 26.1%). Results from our adjusted sensitivity analyses were similar to those from our unadjusted analyses (Table 2).

**Table 2:** Retention and HIV viral suppression at three and six months.

	n	Intervention Group	Control Group	Unadjusted probability difference (95% CI)	Adjusted probability difference (95% CI)^^^
Retention					
3 months	100	46/51 (90.2%)	46/49 (93.9%)	-3.7% (-14.2%, 6.9%)	-1.2% (-12.4%, 10.1%)
6 months	100	41/51 (80.4%)	43/49 (87.8%)	-7.4% (-21.6%, 6.9%)	-5.0% (-19.8%, 9.9%)
Viral suppression (<40 copies/mL) among women retained in the study					
3 months	92	36/46 (78.3%)	34/46 (73.9%)	4.3% (-13.1%, 21.8%)	2.6% (-13.4%, 18.6%)
6 months	84	35/41 (85.4%)	30/43 (69.8%)	15.6% (-1.9%, 33.1%)	11.0% (-5.6%, 27.6%)
Retention with viral suppression					
3 months	100	36/51 (70.6%)	34/49 (69.4%)	1.2% (-16.8%, 19.2%)	1.1% (-16.2%, 18.4%)
6 months	100	35/51 (68.6%)	30/49 (61.2%)	7.4% (-11.3%, 26.1%)	6.7% (-11.8%, 25.4%)

^ Adjusted for continuous measures of log(10) viral load, gestational age (weeks), and number of children, and a binary indicator for financial dependence on partner.

## Discussion

In this pilot study,we demonstrated the feasibility of conducting a combination adherence intervention that empowers pregnant and breastfeeding women and supports them to address the challenges of adherence to ART treatment in pregnancy and postpartum. This interventions support initiatives towards global targets of having 95% on treatment remaining virally suppressed. [25] We noted the high retention rates over time regardless of study arm. This was achieved in both intervention and control arms with women generally being supported by family members and friends throughout the study period [20].

The retention rates were generally high at three and six months at 92% and 84% respectively. High retention is critical to the effectiveness of ART programs and our study has demonstrated that this is achievable even in low-resource settings, especially when innovative approaches are used to sustain retention rates that otherwise may wane with time. [26,27] We also note that three-quarters of women retained in care were virally suppressed at the three- and six-month study visits with good levels of reported adherence. While this intervention was not powered to see differences between the groups, we observed better outcomes among women in the intervention group.

As a pilot study, our overarching purpose was to examine the feasibility of the study approach and intervention that is intended to be used in a larger scale study. [28] We used this pilot study to evaluate the feasibility of recruitment, randomization, retention, assessment procedures, of the iNSC intervention among pregnant and breastfeeding women living with HIV in Malawi. We noted several lessons and practical issues that would be useful in expanding such a trial.

As part of the trial’s feasibility assessment we previously reported, we explored experiences with the iNSC and whether this was acceptable and could be recommended to others [29]. Those results suggested that the majority of the participants reported that the instructions and discussions on iNSC were easy to follow, with most participants reporting a high degree of satisfaction with the iNSC counseling sessions at all study visits. The majority of participants also expressed a desire to receive this intervention again, if available in the future. Qualitative interview data suggested high acceptability and favorable experiences with counselors’ engagement and the intervention generally. [29] Considered alongside these findings, our pilot study suggests this combination adherence intervention holds promise and should be considered for larger scale evaluation.

Our study contributes to a growing body of knowledge that details critical steps along the PMTCT cascade. Our combined measure of (study retention with viral suppression) was feasible to use and reflects the critical role of both kinds of engagement in HIV care. To date, few studies have used viral suppression as an outcome measure for adherence in pregnant and breastfeeding women living with HIV.

In the past, iNSC has been used in trials of men who have sex with men and to support HIV pre-exposure prophylaxis (PrEP). [30] Its adaptation for pregnant and breastfeeding women is novel and noteworthy. In fact, our overall parent trial studied this approach for pregnant and breastfeeding women with and without HIV. [20,31] Our favorable clinical and safety outcomes related to PrEP support for eligible pregnant and breastfeeding women has been reported elsewhere. [32] A key innovation of this work supports the HIV status neutral approach to preventing vertical transmission that considers both groups, a concept that can reduce stigma and health disparities in HIV service delivery. [33,34] Given the unique challenges faced by antenatal and postpartum populations, such broad approaches can provide important opportunities to support antiretroviral drug adherence—whether in the context of treatment (i.e., ART) or prevention (i.e., PrEP).

Despite our study’s strengths, we acknowledge key limitations. First, as a pilot study, this trial was not designed to measure efficacy. While pilot estimates may help to inform future sample size calculations, given its small scope, their imprecision should be carefully considered. Second, we did not report direct measures of adherence, reasoning that HIV viral load is an established surrogate marker for ART efficacy. Nevertheless, given the time intervals between measurements (i.e., three months), greater granularity about adherence behaviors—for example, electronic monitoring or pharmacologic assessments—could enhance this evaluation and other like it. Finally, we recognize that our results may not generalize beyond the eligibility criteria of targeted study population. In this pilot phase, for example, we conservatively excluded individuals who reported recent intimate partner violence to ensure the safety of this behavioral intervention; however, this group is at particular risk for poor adherence and retention outcomes. Such populations should be incorporated into future trials, to ensure that the intervention is efficacious among those in greatest need.

In summary, in this report, we demonstrate promising clinical outcomes in our assessment of a combination adherence package for pregnant and breastfeeding women living with HIV. Such studies provide critical preliminary data to support future exploration, especially when an intervention (like ours) is being adapted for new settings and populations. That this study has demonstrated acceptability, feasibility, and safety—the latter through this current analysis—is encouraging. [29] The data gathered through this study will help to refine the intervention, as well as the design of future studies focused on efficacy.

## Supporting information

S1 File Consort Checklist. (DOCX)
